# Pathologies of hyperfamiliarity in dreams, delusions and déjà vu

**DOI:** 10.3389/fpsyg.2014.00097

**Published:** 2014-02-20

**Authors:** Philip Gerrans

**Affiliations:** Department of Philosophy, University of AdelaideAdelaide, SA, Australia

**Keywords:** delusions of misidentification, deja vu, dreams, hyperfamiliarity, reality testing

## Abstract

The ability to challenge and revise thoughts prompted by anomalous experiences depends on activity in right dorsolateral prefrontal circuitry. When activity in those circuits is absent or compromised subjects are less likely to make this kind of correction. This appears to be the cause of some delusions of misidentification consequent on experiences of hyperfamiliarity for faces. Comparing the way the mind responds to the experience of hyperfamiliarity in different conditions such as delusions, dreams, pathological and non-pathological *déjà vu*, provides a way to understand claims that delusions and dreams are both states characterized by deficient “reality testing.”

## Introduction

The concept of impaired or absent “reality testing” is invoked to explain conditions characterized by persistent inability to detect and correct misrepresentation of aspects of the world (reality). On this model hallucinations result from faulty reality testing in a perceptual modality and false memories result from faulty reality testing in mnemonic mode (Hobson, 1999; Bentall, 2004; Moulin, 2013). Allan Hobson has described some dreams as states in which a capacity for “reality testing” is deactivated by basic neuroregulatory mechanisms.

In my New Orleans dream I *hallucinated*: I saw and heard things that weren't in my bedroom. I was *deluded*: I *believed* that the dream actions *were real* despite gross internal inconsistencies. (Hobson, 1999, p. 5). (my italics)

Clearly Hobson thinks that in this dream he accepted hallucinatory experience at face value, the equivalent of “seeing as believing,” with the crucial difference that in dreams we do not see but hallucinate. One dream theorist describes the experience of dreaming “as if one is immersed in another ‘reality’ entirely of one's own non-volitional, making (see Rechtschaffen, 1997).” The cause of this immersion in another reality is that “altered functioning of the prefrontal cortex may release from reality-filtering or executive constraint an innate human tendency to create stories that organize past, present, and future reality” (Pace-Schott, 2013).

In other words in these episodes of sleep we lose the ability to detect whether or not sensory or perceptual experience represents reality. What is important here is that Pace-Schott suggests that this results from the deactivation of a neural mechanism for Reality-monitoring or, as he calls it, “filtering.”

These ideas have become relevant to characterization of delusion following recent attempts to conceptualize it as a failure of “doxastic reality testing.” Doxastic reality testing refers to the ability to test experiential beliefs for consistency and correctness with background knowledge, thereby establishing whether they correspond to the world as it is, independent of the subject's mind (Hohwy, 2004). It is for reasons like these that dreams and delusions are both described as states in which a capacity for “reality testing” is reduced or absent.

Richard Bentall, who is an advocate of a version of the “reality testing” model of delusion and hallucination, proposes that we think of reality testing on a model of signal detection. Perceptual systems on this view are essentially hypothesis testers: they adopt the best hypothesis about the causes of transduced inputs and that hypothesis becomes a representation of the external world (Bentall, 1990).

Hallucinations arise when faulty hypotheses are generated, and maintained when they are unable to be tested and/or revised. This model is now a substantial research program into the neurobiological basis of predictive coding in many cognitive modalities (See Friston, 2003; Hohwy et al., 2008; Fletcher and Frith, 2009; Corlett et al., 2010; Clark, 2012; Hohwy, 2012) On this view delusions are instances of the same phenomenon as hallucinations at higher levels of cognitive processing. Both involve, hypothesis testing and revision in order to cancel error signals, which arise at lower/earlier stages of cognitive processing. People with delusions form hypotheses that make sense of sensory evidence. These beliefs serve as models of the world, which interpret sensory inputs.

When patients are unable to test or revise false hypotheses, false beliefs persist (Coltheart et al., 2010). When such false beliefs fit the diagnostic criteria for delusion in DSM IV (“false beliefs about external reality caused by incorrect inference… ”) or V they can be understood as delusions caused by faulty “reality testing.” Because DSM criteria are controversial among both practitioners and theorists (note that on the DSM IV definition Hobson is not right to equate dreams with delusions because it is not clear that he was performing any *inference* about experience in his dream), I limit my discussion here to a subclass of well-defined delusions of misidentification (see below). These delusions involve anomalous experiences that have clear analogs in some types of dreams (see below). In delusions those experiences prompt beliefs that are not revised in the face of background knowledge. Quite why those beliefs are not revised remains elusive, although it does seem clear, at least in the cases of delusion I discuss here, that essential prefrontal systems are conspicuously deactivated. Thus, if we could establish how those prefrontal systems enable anomalous experience to be overridden or explained away in alert waking cognition, we would have a potential explanation of the relationship between delusion, dreaming and normal cognition.

One advantage of the reality testing paradigm is that, like other cognitive approaches, it offers the potential to understand a puzzling phenomenon, delusion, whose characterization is theoretically controversial, in terms of a better understood cognitive process, reality testing. The crudest application of the idea would be that those systems deactivated in dreams are the neural implementation of a “reality testing” system. Delusion is a waking state in which those systems are also hypoactive or compromised.

Unfortunately, as Bentall himself notes, reality testing itself is a term of art. It combines an intuitive definition of what is going on in delusion (people form and maintain beliefs which are at odds with reality) with formalization, in the form of Bayesian inference, which allows for experimental manipulation. As he says: there is no reason to believe that perceptual states have the labels “real” or “imaginary” printed on them. We have to infer how best to describe any particular experience from whatever information there is available to us, and there is no unique source of information that we can rely on to make this judgment (Bentall, 2004, p. 364).

Bentall is right that there is no unique source of information that could tell us whether a representation is “real” or not. Nor, *ex hypothesi* is there a unique cognitive process evolved or developed to detect those labels. Thus, the concept of “reality testing” used in this way is unconstrained. It is by no means clear

- that the mind has evolved a cognitive system specialized for testing representations for “reality” rather than, for example, congruence with domain specific internal models. In the case of some non-veridical memory experiences, for example, subjects may feel as if they are remembering a previous experience. This phenomenon of feeling as though an experience is a memory is baptized “*déjà vécu*” (Moulin, 2013). This arises from a faulty signal of congruence between a current representation and a previously constructed one rather than a signal that a memory is “real.”
- that such a capacity (mal)functions in the same way across cognitive modalities (perception, memory, belief) *and* in delusions and dreams. Nonetheless in this paper I cautiously pursue the idea that Bentall and Hobson are on the right track. Phenomenological similarities between (some) delusions and dreams, combined with some similarities in patterns of global neural activation and deactivation support the idea that delusions and dreams may have some important cognitive properties in common (Schredl and Hofmann, 2003; Noreika, 2011; Windt and Noreika, 2011). Not only that, but those cognitive properties may underwrite capacities for what is loosely described as reality testing. As John Nash explained his delusions to an interviewer “it's kind of like a dream. In a dream it's typical not to be rational” (Nash, 2002) I think Nash intended to characterize the similarity between dreams and delusions, not in terms of a reasoning deficit (after all Nash remained capable of high-level mathematical and logical reasoning while delusional), but in terms of an *absence* of reasoning about experiences and thoughts which would in neurotypical waking cognition prompt some kind of metacognitive response.

Clearly delusions are not simply waking dreams, nor are the experiences that prompt them simply accepted without metacognitive evaluation (this is also true of some dreams in which people are aware of the anomalous nature of experience). Nonetheless some delusions do seem to involve abnormalities in the way people respond to experiences whose content would typically be detected and rejected as anomalous. An instructive comparison is provided by *déjà* or *jamais vu* (Sno, 2000). Such experiences, which I will characterize as the experience of hyper-and hypofamiliarity for places (see below), are relatively common and may even trigger thoughts such as “I've (never) been here before”. However, such thoughts are not typically elaborated or incorporated into the subject's belief system. If, however, a subject of a *déjà vu* experience continued to maintain that she had previously visited the place in question and even elaborated that belief, confabulating corroborative detail, she might attract a diagnosis of delusion of misidentification for place. She would have an intractable false belief, based on faulty inference about the cause of her experience (in this cases hyperfamiliarity), namely, that her experience of familiarity was based on veridical recognition. Such cases have been baptized Recollective Confabulation by Moulin (2013; O'Connor et al., 2010).

There is evidence that ability to correct or override these kinds of thoughts triggered by experience depends on activity in right dorsolateral prefrontal circuitry. When activity in those circuits is absent or compromised subjects are less likely to challenge and revise thoughts prompted by anomalous experiences and may adopt them as beliefs (Gilbert et al., 2010; Barbey and Patterson, 2011; Barbey et al., 2013). This hypothesis fits the evidence about the cause of some delusions of misidentification characterized by anomalies of familiarity consequent on lesion to the right fusiform gyrus (a circuit known to be implicated in face recognition) accompanied by lesion to right dorsolateral prefrontal circuitry. Within a classic neuropsychological framework this suggests that an erroneous signal of (hypo- or hyper-) familiarity for a seen face generates a belief which cannot be corrected due to hypoactivity in the dorsolaterally-regulated systems (Papageorgiou et al., 2003, 2005).

Does this suggest that our capacity to reject thoughts triggered by such experiences depends on a capacity for “reality testing” part of whose neural substrate is dorsolateral prefrontal circuitry? I shall argue that this is not *quite* the right way to explain the difference between the delusional and non-delusional response. An important part of my argument is consideration of the way in which anomalous experiences of familiarity arise and are processed (or not processed) metacognitively in dreams. Triangulating between the three cases, dreams, delusions and alert waking cognition allows us to deconstruct the concept of “reality testing.” There is of course an important *caveat* to be entered here: hyperfamiliarity for places and faces and the responses they engender in dreams, delusions and alert waking cognition are different cognitive phenomena. Nonetheless there is a consensus, on which I draw, that experience of familiarity for faces and places depends on similar cognitive processes (O'Connor and Moulin, 2010; O'Connor et al., 2010). In addition some dream theorists have explicitly made the connection between hyperfamiliarity for faces in dreams and delusions based on the nature of cognitive processing in face and place recognition (Revonsuo and Tarkko, 2002). Thus, I will assume that we can draw some interesting conclusions about the way in which anomalous experiences are dealt with by looking at differences in the way the mind responds to anomalies of hyperfamiliarity in dreams, delusions and non-delusional waking cognition.

## Hyper- and hypofamiliarity

In delusions of misidentification people report seeing “doubles,” imposters, people in disguise, people changing appearance and identity (Ellis, 1998; Breen et al., 2000; Ellis and Lewis, 2001). The phenomenology of these delusions can be explained in terms of abnormalities of face-processing that produce a representation in which elements normally bound together such as face, name, autonomic response to a familiar person, and identity may dissociate (Revonsuo and Salmivalli, 1995; Revonsuo and Tarkko, 2002; Noreika et al., 2010a). For example if a representation of the facial appearance of a spouse is not bound to information about familiarity which drives autonomic response the result will be an incongruity: the patient sees a person who appears familiar but she does not have a characteristic autonomic reaction to that person. This incongruity, produced by the failure to bind the familiar face to a signal initiating autonomic response, leads to the delusional response: “my wife has been replaced by an imposter.” This is the Capgras delusion, which explains the experience of hypofamiliarity for a familiar face. Another delusion of misidentification is the Fregoli delusion in which people say that they are being followed by a familiar person in disguise (de Pauw et al., 1987; Joseph and O'Leary, 1987; Eva and Perry, 1993; Ellis et al., 1994; Wolfe and McKenzie, 1994; Ellis and Szulecka, 1996). In this case the patient sees a stranger and does not recognize her, but experiences the feeling of familiarity. The Fregoli delusion is generated to explain this mismatch between feeling as though the seen person is familiar combined with inability to recognize them. Thus, these delusions are *responses* to the experience of hypofamiliarity and hyperfamiliarity respectively.

The standard account of these responses is that they are empirical beliefs, that is beliefs adopted to explain experience (Maher and Ross, 1984). The key *explanandum* then becomes the difference between the way delusional and non-delusional minds respond to anomalous experiences whose content is inconsistent with background knowledge.

Dream experience becomes relevant to this issue because many dreams involve experiences that are highly inconsistent with background knowledge. Often, however, in dreams (at least of the type discussed by Hobson above) no background knowledge is brought to bear and experiences are not explained or explained away. Most dream theorists explain this in terms of deactivation of neural circuitry required for the necessary metacognitive processing (Solms, 2000; Gottesmann, 2010; Domhoff, 2011). Their focus then becomes clarifying the nature of that processing and the way neural mechanisms implement it. A crucial part of that project is comparing metacognitive function in different modes of waking, sleeping and dreaming. Bizarre experiences of misidentification occur in dreams (Revonsuo and Salmivalli, 1995; Röhrenbach and Landis, 1995; Kahn et al., 2002; Revonsuo and Tarkko, 2002; Schwartz and Maquet, 2002; Desseilles et al., 2011). Revonsuo and Tarkko (2002) analyzed 592 dream reports that contained bizarre dream characters (people who appear in dreams). The authors noted that inappropriate feelings of familiarity are one of the most commonly reported bizarre dream experiences and explained their occurrence as follows: “we may interpret the abundance of pseudo-familiar persons in dreams as over-activation of face recognition units: they fire even when the face percept does not correspond to any of the descriptions in the store. This creates the inappropriate feeling of familiarity” (Revonsuo and Tarkko, 2002; Schwartz and Maquet, 2002).

Schwartz and Maquet (2002) note in their discussion of dream experience that it includes similarities to many other cases characteristic of neuropsychological disorder such as achromatopsia (inability to perceive color) and polyopsia (perception of multiple images of a single object). These cases can be explained in terms of abnormal patterns of activity produced when later stage of processing in the ventral visual stream are driven by subcortical afferents rather than its usual inputs from early vision. The result is bizarre juxtaposition of representational elements whose assembly into a coherent representation is normally driven by early perceptual processing.

Crucially one of those elements is the feeling of familiarity. There are different explanations of the nature of this feeling. On one view, popular in the face recognition literature, the feeling is an affective one consequent on the process of identification. These models give a prominent role to the amygdala in generating that feeling, postulating disturbances to connectivity between the face recognition circuitry and downstream affective processing.

Interestingly, (see below), this view receives some support from the literature on *déjà vu* which notes many cases of persistent and unfocused *déjà vu* in temporal lobe epilepsy. In these cases the feeling is persistent and pervasive and everything feels strangely “familiar” (Illman et al., 2012).

Another view describes the feeling as an instance of higher order “cognitive feelings” (O'Connor et al., 2010),that is as a distinctive form of phenomenology, generated by process of recognition, which does not necessarily involve downstream affective processing. It is worth mentioning that the “cognitive feeling” interpretation is most strongly argued for another type of case, *déjà vecu*, in which people report genuinely remembering episodes of experience that could not have occurred. These cases are distinguished from *déjà* vu (driven by perceptual recognition) because the relevant signal, generated in the process of mnemonic retrieval, informs the subject that the information retrieved is familiar (O'Connor et al., 2010).

Slightly different juxtapositions of identifying information in dreams have been remarked on by Schwartz and Maquet (Röhrenbach and Landis, 1995; Schwartz and Maquet, 2002, p. 26). They use the following examples. “I had a talk with your colleague but she looked differently, much younger, like someone I went to school with, perhaps a 13 year old girl.” In another case a subject reported “I recognize A's sister. I am surprised by her beard. She looks much more like a man, with a beard and a big nose” Schwarz and Maquet describe these as Fregoli-like phenomena generated by activation in the facial fusiform area.

An interesting feature of these reports is that the mismatch here is between facial appearance and identity (“your sister”) rather than facial appearance and a feeling of familiarity. In this sense these cases resemble the phenomenon described by Revonsuo and Tarkko (2002) as appearing infrequently in dreams “cases of imposter relatives; persons we have never met in the real world but who we accept as our ‘sisters’ ‘brothers’ or ‘cousins’ or ‘uncles’ in the dream.”

The binding hypothesis suggests that representations of appearance and identity are bound to signals of familiarity and that the binding process can go awry in different ways, generating different forms of misbinding between identity, appearance and familiarity. The binding hypothesis suggests that the processing involved in recognition of a familiar face generates a signal of familiarity that produces (directly or via the activation of downstream affective processes) feelings of familiarity, which can, in anomalous cases, generate feelings of hyperfamiliarity. The binding hypothesis also explains the nature of the anomalous experience characteristic of Fregoli and Capgras delusions. They involve the experience of familiarity for unfamiliar faces and unfamiliarity for familiar faces respectively.

Some recent work on *déjà vu* provides a further basis for the idea that feelings of familiarity can dissociate from recognition and that this dissociation can lead to delusions of misrecognition. We noted above that while fleeting experience of *déjà vu* is common associated delusions are rare: we rarely encounter someone with the delusion that they have previously visited an unfamiliar house. Yet clinicians encounter something very like such a delusion in recollective confabulation (RC). In recollective confabulation a subject experiences something like *déjà vu* for a place or event and then explains that experience by constructing an account of a previous encounter to fit with that feeling of familiarity (Moulin, 2013).

An important point to note about recollective confabulation is that it is initiated by feelings of familiarity for a novel object rather than the process of recollection *per se*. The patients in question have relatively intact recollective processes in standard tests, and Alzheimer's patients with recollective problems do not necessarily have *déjà vu or* recollective confabulation (Moulin, 2013).

The hypothesis of Moulin (2013) is that when we recognize or recall a familiar event very early processes generate a signal of familiarity and that signal initiates subsequent processes of representing the event in detail: “a fast familiarity response guides retrieval processes until recollection provides a subjective feeling of remembering and additional contextual information” (p. 1551). At a subsequent stage the filling in of details becomes explicit and analytic. Thus, “RC would not be caused by a deficit in recollection *per se* but the use of somewhat intact recollection processes to justify feelings of familiarity” (p. 1542).

*Déjà vu*-type experiences occur when feelings of familiarity are triggered incorrectly. The patient is then in a situation of experiencing familiarity, which normally drives recollection and further elaboration of contextual detail. The patient proceeds to this stage, producing the recollective confabulation.

Moulin (2013) explicitly draws an analogy between this process and the generation of delusions of misidentification for (un) familiar faces. “Critically, delusional misidentifications or people, places and time, have been hypothesized as resulting from memory-like disruptions to feelings of familiarity.” Following Feinberg and Shapiro he describes these delusions as “an illogical attempt—a confabulation- of why ‘the familiar is experienced as strange or vice versa”’ (p. 46, 1542).

## Metacognitive failure

Moulin like many theorists notes that that *déjà vu*, even persistent *déjà vu*, does not normally lead to the type of false belief generated by recollective confabulation. Confabulation and delusion are not easy to distinguish. On some views they are very closely related. Moulin for example *equates* delusions of misidentification and reflective confabulation: “the patient will justify this mismatch with the belief [Capgras delusion].” And reflective confabulation involves the use of “intact recollective processes to justify feelings of familiarity” (p. 1542).

Confabulation is the process of generating a story consistent with experience, which is not constrained by epistemic norms of consistency and coherence. As long as the confabulation “fits” the experience it is adopted and sometimes elaborated irrespective of whether or not it is consistent with background knowledge. One interesting thing about confabulation is that it often coexists with intact capacities for reasoning on topics other than the confabulated belief.

The equation of confabulation and delusion is most persuasive in cases of beliefs generated as a response to experiences of pathological familiarity, because such beliefs often arise in cases whose cognitive structure resembles the pattern of deficits in classic confabulation: a deficit in a perceptual system (in this case a face or place recognition) combined with a deficit in right prefrontal systems. The first generates the relevant experience and the second makes it impossible to override a confabulatory response to that experience.

For example Moulin explains the persistence of the confabulation as a consequence of a metacognitive deficit that produces “the failure to *metacognitively oppose* that evaluation” (p. 1549).

Thus, Moulin's discussion of reflective confabulation for *déjà vu* suggests a cognitive structure for dealing with anomalous experience. The experience is initially dealt with by systems that “justify” the experience. Crucially this justification is responsive *only to the experience itself*: the delusions of misidentification and reflective confabulation for pathological *déjà vu* provide a belief adequate to the feeling of hyperfamiliarity for face or location. That belief however is inconsistent with readily available general knowledge: imposters and previous acquaintance with strange locations are highly unlikely. In the normal course of events general knowledge triumphs. But somehow the delusion/confabulation is not overridden by background knowledge.

This suggests that in the normal course of events we rapidly construct a justification for anomalous experience adequate only to the experience itself, which is overridden if it turns out to be inconsistent with background knowledge. Ultimately we adopt an hypothesis that fits the anomalous experience into an epistemic framework. A sudden headache prompts the thought “I have brain cancer” but we almost instantaneously challenge that belief and substitute it with another (“my skateboard accident”, “my cage fighting audition”) drawn from a wider epistemic context. It is that second step which is unavailable to the deluded in these cases.

An important cause of that unavailability seems to be hypoactivity or lesion to right dorsolateral prefrontal areas. Moulin locates these deficits as crucial to the metacognitive failure involved in recollective confabulation.

Crucially similar deficits have also been observed in delusions of misidentification. Papageorgeiou et al. studied nine patients with delusions of misidentification, Capgras, Fregoli, Intermetamorphosis, with some patients suffering more than one of these delusions (Papageorgiou et al., 2003, 2005). The 2005 study measured event-related potential (ERP) focusing on the P300 ERP component. The delusional patients group showed significant reductions in P300 amplitude in frontal areas of the right hemisphere compared to controls.

The conclusion that hypoactivity of the frontal lobe of the right hemisphere is involved in delusion is consistent with other neuropsychological studies. For example in a group of 33 patients with Alzheimer's disease, of whom 18 had a content specific delusion concerning place, person or object, Single Positron Emission Topography revealed hypoperfusion in frontal lobe of the right hemisphere in the delusional groups compared to the 15 non-delusional sufferers (Coltheart, 2007).

These and other cases lead Coltheart to conclude that it is specifically deficits in the frontal lobe of the right hemisphere that are the cause of the failure to challenge the delusional belief in delusions of misidentification. He describes those circuits as the substrate for a system of “belief evaluation” which reconciles candidate beliefs with background knowledge according to Bayesian principles of abductive inference. When they are lesioned or hypoactive the patient remains hostage *not to experience* but to a belief adequate only to experience, not the wider epistemic context.

I endorse this account but I think that it leaves out something that is hinted at but underdescribed in Moulin's account, namely the *origin* of the candidate belief that survives as recollective confabulation or delusion. The belief for example that “I have been here before” or “I am being followed by a stranger in disguise,” while it is adequate to the experience, does not simply *report* an experience of hyperfamiliarity.

It provides a *context* that makes it intelligible. The crucial question is why this process of contextualization does not seem to incorporate relevant background knowledge, including other beliefs about likely causes, and the implausibility of the delusion. The patient's belief is just about the most *unlikely* empirical hypothesis. This is why the DSM IV defined delusion as “a false belief about external reality based on incorrect inference.”

The cognitive relationship between delusions and dreams helps explain the exclusion of background knowledge from this process of contextualization. Dreams are produced by activation in a neural system, the default mode network, which provides the raw materials for confabulations and delusions as well as narrative contexts for salient experience in waking cognition. When we compare the activities of the default mode network in different conditions we can see why the delusional patient does not override her implausible belief.

## The default mode network

Unlike other animals we can re-experience the past (episodic memory) in order to preexperience the future (Suddendorf and Corballis, 2007; Botzung et al., 2008). In order to do this we simulate experiences, running the relevant neural mechanisms offline. This role of simulation in planning provides a new, unifying, interpretation of the connection between episodic memory (recollection) and prospection as aspects of a unified capacity for stimulus-independent thought (Schacter et al., 2000, 2007, 2008). This view is supported by evidence that damage to neural circuitry known to be essential for episodic memory also impairs imagination (Hassabis and Maguire, 2007, 2009; Hassabis et al., 2007; Maguire et al., 2010; Maguire and Hassabis, 2011). This simulatory capacity employed in the service of planning is now baptized *mental time travel* to capture the fact that it allows us to escape the stimulus-bound present, review the past and preview the future by projecting ourselves into different scenarios. Basically it allows us to simulate alternative personal histories. This type of simulation provides the subject with a subjective, highly emotionally inflected, representation of the world based in her own experiential history and imaginative capacities (Smallwood and Schooler, 2006; Christoff et al., 2009). This *subjectivity* is, of course, vital for action: if our plans were not inflected by emotional and motivational states and were not biased to the first-personal perspective they would not be useful guides to action. In deciding whether to remarry or change jobs one's own life experience is what matters. The fact that someone else would enjoy being married to X or working in a certain job is only incidentally relevant.

The relevant circuitry which enables us to recall and rehearse experiences is (slightly misleadingly) named the default mode network (DMN) because its activities are detected in a variety of “resting” scenarios as well as during active mental time travel (Mason et al., 2007; Buckner et al., 2008; Broyd et al., 2009). Thus, DMN activity is observed, not only in memory, imagination and planning, but also in daydreaming and mindwandering as it is known: states of reverie in which narrative fragments combine and recombine semi-spontaneously. The best interpretation of these phenomena I suggest is that when the DMN is not actively controlled during a specific task of building a context for action (imagining the torture of attending another committee meeting on “online pedagogy” as a prelude to resigning from the university) it continues to run in what amounts to a screensaver mode. Running in this way it is available to be triggered to provide a context for any novel information referred by perceptual processes or to provide narrative scenarios as solutions to longer-term problems.

The fact that we call undirected activity in the default network daydreaming is revealing because another conditions in which the DMN is active is dreaming. As Domhoff puts it:

the likely neural substrate that supports dreaming, which was discovered through converging lesion and neuroimaging studies, may be a subsystem of the waking default network, which is active during mind wandering, daydreaming, and simulation… If this theory is correct, then dreaming may be the quintessential cognitive simulation (2011 1163). See also Fox et al. (2013)

There are a variety of conditions in which the DMN is engaged. One is planning which combines experiential memory and imagination, which often go together: we review the past to preview the future. This is a condition in which the default system is recruited for a specific goal: the evaluation of actual and possible experience relevant to a specific outcome or event. Effectively we rehearse a mini-narrative or episode with ourselves as the central character. In cases where we empathize or solve social problems by simulating other's experience the DMN is also engaged, consistent with the hypothesis that some aspects of social cognition involve personal simulation (Mitchell et al., 2005; Klein, 2011; Reniers et al., 2012). However, the DMN is also relatively active in daydreaming, mind wandering and unfocussed rumination. As Broyd and collaborators put it: the properties of the default network reflect “low frequency toggling between a task-independent self-referential and introspective state [daydreaming] and an exteroceptive state that ensures the individual is alert and attentive to unexpected or novel environmental events.” (Broyd et al., 2009, p. 281).

This suggests a role for the DMN consistent with theories of cognitive architecture that treat it as an hierarchical error correction system. The basic idea is that higher levels of cognitive control are activated by signals of discrepancy or error which cannot be resolved at lower levels. Anomalous experiences in effect represent problems which cannot be solved at lower levels by perceptual and sensory systems. Higher levels of executive control then are engaged to resolve the problem (Fletcher and Frith, 2009; Clark, 2012).

The first stage is to provide an autobiographical context for the experience: to rehearse scenarios in which such experiences have or could occur as a way of gathering information relevant to responding. The so called DMN is essentially a mechanism which evolved to provide these kinds of actual and possible contexts for experiences.

This interpretation is supported by considering the tasks for which the DMN is not recruited. When the mind is directing perceptual attention (focused on features of the environment), solving abstract problems or reevaluating goals and strategies the default network is not engaged. As Whitfield-Gabrieli and Ford put it: the “DMN in the healthy brain is associated with stimulus-independent thought and self-reflection and … greater suppression of the DMN is associated with better performance on attention-demanding tasks” (Whitfield-Gabrieli and Ford, 2012, p. 49).

The DMN is essentially a mechanism for constructing the elements of personal narratives: stories and s which make experience intelligible in terms of goals and motives. As Pace-Schott describes it the DMN is the substrate of a capacity “to represent reality in the form of narrative—a ‘story-telling’ instinct or module” (Pace-Schott, 2013). Thus, we can picture the DMN as a mechanism which provides a necessary subjective perspective on experiences, locating them in personally and or socially compelling narratives. The same information can also be represented neutrally and impartially as pure description or theoretical explanation, but such representations are processed by different cognitive systems. The processes which accomplish these forms of representation are baptized *decontextualized* processes by cognitive psychologists.

In fact there is evidence that the DMN needs to be *deactivated* for these kinds of *decontextualized* cognitive processes. Subjects (including delusional subjects) who have hyperactive or hyperassociative DMN perform poorly at tasks which require decontextualization such as symbolic reasoning and memory tasks (Broyd et al., 2009; Whitfield-Gabrieli et al., 2011). A crucial hub of decontextualized processing is the right dorsolateral prefrontal cortex.

These facts help clarify the nature of the “metacognitive deficit” identified by Moulin in his cases of recollective confabulation and delusions of misidentification. An hypothesis which fits the evidence is that these patients have the anomalous experience of hyperfamiliarity for places or faces. That experience is in effect an error signal to higher-order cognitive processes that something unpredicted has occurred. The mind's initial response is to build a narrative context, a story, which fits the experience and locates the subject in relation to it. “I've been here before… ” “A familiar person disguised as a stranger is following me.” Such responses are quite natural as fleeting thoughts occasioned by instances of *déjà vu* remind us.

However, as such cases also reminds us, we have the capacity to correct or override these stories. But in order to do so we need a fully functioning capacity for decontextualization which allows us to treat the story element as an hypothesis to be disconfirmed or confirmed by evidence, rather than a narrative element which provides an intentional interpretation of an experience.

However, as we saw in the classic cases of pathological *déjà vu* and recollective confabulation discussed by Moulin and the cases of delusional misidentification discussed by Papageorgeiou this type of decontextualization depends on activity in right dorsolateral prefrontal circuitry. Where those circuits are lesioned or hypoactive the patient seems unable to override the delusional or confabultory hypothesis. It is worth noting although I do not discuss it here that the relevant hypoactivity may in some other cases of delusion be *relative* to activity in the DMN rather than absolute as in lesions. But lesions to the rDLPFC, in combination with lesions to right frontal areas involved in recognition provide the purest cases of anomalous experience combined with inability to decontextualize. In such cases patients are cognitive hostages to their narrative impulse.

Should we describe someone unable to override a thought generated as a personal narrative context for an experience as suffering from problems with “reality testing”? Coltheart (2007) describes such people (although he does not specify the mechanism which generates the thought) as having a deficit in “belief evaluation” by which he means Bayesian confirmation. Moulin calls it a deficit in metacognition involved in “challenging” the relevant thought.

My view is that the condition is sufficiently well described when we identify the cognitive nature of default processes and the decontextualized processes which normally supervise them. It is at this point that the nature of dream experience becomes relevant once again. Not only are some dreams characterized by the experience of hyperfamiliarity but they are also characterized by activity in the default network accompanied by deactivation of the rDLPFC.

## Dreams

Dreams are not a monolithic cognitive phenomenon. They include terrifying fully immersive nightmares as well as lucid dreams, in which the dreamer is aware of the fact that she is dreaming (Blagrove and Hartnell, 2000; LaBerge, 2007; Hobson, 2009a,b; Noreika et al., 2010b; Spoormaker et al., 2010); non-rapid eye movement (NREM) dreaming, often characterized by high degree of coherence and resemblance to actual, often stereotypical, scenarios; and the kinds of immersive and sometimes bizarre dream experiences described by Hobson in which the subject is unaware that she is dreaming and seems to accept anomalies of experience that would in waking cognition attract explanation.

It is this last type in which we are interested. They occur in rapid eye movement (REM) sleep, a state characterized by patterns of activity in the DMN that resemble activity during wakefulness (Solms, 2000; Domhoff, 2005; LaBerge, 2007). Importantly, during REM sleep the DMN is not communicating with areas responsible for other cognitive processes. During REM sleep, for example the mind is relatively disengaged from the perceivable environment, and the rDLPRC and some medial prefrontal regions are conspicuously deactivated (Schwartz and Maquet, 2002; Hobson, 2009a,b; Nir and Tononi, 2010).

Thus, dreams represent an extreme case of hyperassociative default processing, “a unique and more fully developed form of mind wandering, and therefore … the quintessential cognitive simulation” (Domhoff, 2011, p. 1172). Subcortical inputs to the default system in dreams come from limbic and dopaminergic systems. The latter is the mind's salience system, which evolved to make some representations highly salient, making them pop out of the cognitive background, command attention and engage motivational systems. Thus, in some REM dreams the default system churns through representations of scenes which can feel significant and emotionally charged (Hobson, 1999; Solms, 2007; Dawson and Conduit, 2011; Desseilles et al., 2011). The connection between dreams and delusions noted on phenomenological grounds by many patients and clinicians and on neurobiological grounds can thus be explained in neurocognitive terms. REM dreams are characterized by DMN activity where the default system is not playing its waking functional role of providing an autobiographical narrative context for salient experiences. Rather it seems to be manufacturing story fragments and scenarios whose patterns of association reflect the way activation propagates in a disinhibited DMN.

Gottesman puts things this way “the dorsolateral prefrontal deactivation observed both during REM sleep and in schizophrenia seems to suppress or decrease its own functions, including the loss or decrease of reflectiveness, and at the same time disinhibits older subcortical structures and corresponding functions, with the exaggeration of accumbens' and amygdala nuclei's own processes: in our case, the appearance of hallucinations, delusions, bizarre thought processes, and affective disturbances (Gottesmann, 2006).

Clearly the causal route to reduced rDLPFC activity is different in the cases of dreaming, schizophrenic psychosis and the delusions of misidentification we discuss. In dreams this deactivation of the rDLPFC and relative hyperactivation of the default network is a result of changes in the balance of cholinergic and aminergic regulation of the brain. In the Delusional Misidentification Syndromes described by Papageorgeiou (and in typical cases of confabulation) there is lesion to the rDLPFC. In schizophrenia relative hyperactivity of the DMN is the product of a complex set of interactions between different brain systems. A common factor is the reduction or absence of DLPFC activity.

Precisely how to characterize cognitively the dysregulated activity of the DMN in REM dreams remains an open question. Hobson treats it as the equivalent of psychosis. Pace-Schott has proposed that REM dreams are in fact stories, or represent the activity of a story-telling system disconnected from executive control. “Therefore, in both confabulation and dreaming, altered functioning of the prefrontal cortex may release from reality-filtering or executive constraint an innate human tendency to create stories that organize past, present, and future reality. Dreaming may represent a potent, naturally occurring form of confabulation in which imaginary events are not only created and believed but are vividly experienced as organized, multimodal hallucinations” (Hobson, 1999; Pace-Schott and Hobson, 2007; Pace-Schott, 2013).

So although activity across the brain is different in the two cases of delusion and REM dreaming an important similarity between dreams and delusions is reduced or absent activity in the rDLPFC which leaves the subject “at the mercy” of the DMN.

## Conclusion

Some theorists and patients have assimilated delusions, qua psychotic states, to dreams on the basis of phenomenology. Others have noted similarities in the neurobiological profile of psychotic and delusional patients. Clearly delusions are not simply waking dreams and, in any case, both delusions and dreams are heterogeneous phenomena. Nonetheless there is an intuition here worth pursuing. That intuition turns out to be reflected in terms of the cognitive properties of the default mode network. The default mode network is essentially a cognitive system that produces story elements or fragments that allow a subject to situate experiences in a narrative context. Dreams reflect the operations of that system *relatively* disconnected from both the external world and from higher-level cognitive supervision. Delusions are produced by activities in the default system in a waking state which has a number of cognitive biases introduced at different levels of cognitive processing. Accurately describing the operations of the default mode network and its interactions with other systems on a case-by-case basis in both delusions and dreams is a viable research program. Here I have tried to begin the project by focusing on the interactions of the default mode network and the face recognition system in delusional, dreaming and neurotypical waking states characterized by the experience of “hyperfamiliarity.” The Table 1 below describes the basic cognitive properties of each state and the way hyperfamiliarity arises and is dealt with in each case.

**Table 1:** **Summarizing cognitive similarities and differences between dreams and delusions**.

**Cognitive mode**	**Wake**	**NREM**	**REM**	**Delusion (e.g. Fregoli consequent on hyperfamiliarity)**
Automatic processing	Acquisition and manipulation of information	Iteration of information	Endogenously driven associative processing	Anomalies of experience become hypersalient
Face processing	Binding driven by perception Fleeting hyperfamilarity (déjà vu)	Intact percepts	Binding elements dissociate (hyperfamiliarity)	Sustained hyperfamiliarity
Default though	Subjective context for salient experience	Rehearses stereotyped narrative fragments	Incoherent narrative fragments	Hyperactive, not regulated by decontextualized processes
Decontextualized thought	Reality testing, metacognition	Deactivated	Deactivated	Hypoactive or inaccessible for delusional content
**PHENOMENOLOGY**				
Perception	External vivid	Dull or absent	Internal vivid	Sustained hypersalience may be discrete or global.
Movement	Continuous voluntary	Episodic involuntary	Commanded but inhibited	Voluntary
Thought	Logical, coherent progressive	Logical, coherent perseverative	Incoherent, associative	Focused on delusional topics and narrative associations. Absent reality testing
**NEUROCHEMISTRY**				
5HT (serotonin)	High	Decreasing at end of cycle	Low	
NA(noradrenaline)				
Acetylcholine	Low	Increasing at end of cycle	High	
DA (dopamine)	Salience within a functioning control hierarchy	Tonic, stabilises patterns of representation	Phasic, modulates turmover of representations	Effect of neurotransmitter abnormality in delusion is to up regulate default system. Varies from case to case. Established for schizophrenia. Produces sustained hypersalience, global or local

Schwartz and Maquet (2002, p. 29) point out that it is characteristic of bizarre experiences in REM dreams that quite incongruous experiences are accepted “without much surprise.” Their choice of words is quite apposite within the increasingly influential Bayesian framework for understanding the mind. Within that framework the technical term given to error signals is “surprisal” since they signal that information inconsistent with a prior model of a cognitive domain cannot be accommodated by the relevant cognitive system.

The experience of hyperfamiliarity characteristic of delusions of misidentification is an instance of the detection of surprisal. It is a case of a signal of familiarity mismatched to an unfamiliar face. In a delusional mind the DMN performs its normal role, generating a narrative fragment, which is subjectively adequate to the experience. By this I mean it provides a context for it that makes it intelligible in terms of agency and intention. The process is similar to that involved in thinking “I've been here before” consequent on a déjà vu experience. Similar experiences and responses occur in classes of REM dreams characterized by activity in the DMN.

Whether we describe such thoughts in waking cognition, when maintained in the face of contrary background beliefs, as delusions or confabulations, a crucial factor in their maintenance seems to be reduced activity in the rDLPFC. When the rDLPFC is hypo or inactive the mind is hostage to the functioning of the DMN.

Pace-Schott (2013) has a distinctive account of DMN functioning (that the dreaming mind in REM sleep is a mind in story telling mode) whose details do not matter to the key point. The DMN is necessary for the construction (and no doubt the consumption) of narrative. In essence the DMN constructs representations of events and scenes which treat experiences as elements in a story. In order to construct a coherent story the default network needs a degree of supervision: the story needs a goal or endpoint. That goal might be a future action or a current event depending on whether the DMN is providing context for a planned or actual experience. In REM dreams of the type discussed here there is no current context for the experience: the mind is not engaged in planning, inference, decision making or engaging with the environment via perceptual and sensory systems. Hence the normal patterns of cognitive processing are suspended. In particular, anomalies and incongruities, which would normally initiate a train of error detection and correction, do not activate any metacognitive responses. The situation is different in lucid dreams (in which situations may be recognized as bizarre) or intermediate cases in which dreams rehearse segments of coherent waking life. Nonetheless it does remain striking that dream reports almost never include episodes of focused, sustained, metacognitive problem solving.

Such facts merely confirm some ideas developing within dream theory: dreams are not all-or-nothing states of representational chaos. They reflect the mind's structure and operations under a range of different conditions. In one such condition which generates bizarre experiences of hyperfamiliarity the DMN is relatively uncoupled from other systems with which it normally cooperates and is endogenously activated by subcortical inputs. If Pace-Schott is correct then the DMN in this condition generates story fragments and episodes which reflect disinhibited patterns of association.

The hypothesis presented here is that delusions arise when experiences trigger activity in a cognitive system, the DMN, that evolved to manufacture story elements: the raw material of autobiographical narratives. Delusional thoughts survive in the mind when those narrative fragments are not treated as descriptions of reality or hypotheses or about the causes of experience. The causes of this insulation, which may not be absolute, vary from case to case but the evidence from dreaming supports the idea that a crucial neurocognitive system recruits the rDLPFC.

### Conflict of interest statement

The author declares that the research was conducted in the absence of any commercial or financial relationships that could be construed as a potential conflict of interest.
